# Catch of *Reesa vespulae* in Heritage Environments

**DOI:** 10.3390/insects15060405

**Published:** 2024-06-01

**Authors:** Peter Brimblecombe, Pascal Querner

**Affiliations:** 1 Department of Marine Environment and Engineering, National Sun Yat-sen University, Kaohsiung 80424, Taiwan; 2 School of Environmental Sciences, University of East Anglia, Norwich NR4 7TJ, UK; 3 Natural History Museum Vienna, Burgring 7, 1010 Vienna, Austria; pascal.querner@nhm-wien.ac.at; 4 Department of Integrative Biology and Biodiversity Research, Institute of Zoology, University of Natural Resources and Life Sciences, Gregor-Mendel-Straße 33, 1180 Vienna, Austria

**Keywords:** parthenogenesis, skin beetle, trapping, museum pests, IPM, Austria

## Abstract

**Simple Summary:**

The skin beetle *Reesa vespulae* is regularly found beyond North America where it originated, having arrived in Europe in the mid-20th century. Initially associated with stored food products, the beetle causes damage in museums by attacking hides, furs, dried plants and zoological collections. Although still only found in a small fraction of museums, it is occasionally present in large numbers. A single female can continue to reproduce, meaning this species can persist over long periods of time. Larvae are trapped more frequently during infestations, suggesting *R. vespulae* may range widely in search of food.

**Abstract:**

The skin beetle *Reesa vespulae* is regularly found beyond North America where it originated. The larvae cause considerable concern in museums, as they damage hides or furs in addition to being a special source of damage to collections of dried plants in herbaria or collections of insects and other zoological specimens. *Reesa vespulae* arrived in Europe in the mid-20th century and was associated mostly with stored food products, but over time, it has become recognised as a museum pest. Although it is still uncommon and may only be observed in a small fraction of museums, when the insect is found in large numbers, it can cause problems. Catches from blunder traps in Austrian museums and from an online database in the UK were used to track changing concern over the insect. As a single female beetle can continue to reproduce because the species is parthenogenetic, its presence can persist over long periods of time. Although small populations in museums are typically found in the adult form, the larval forms are more common where a site is infested by high numbers, perhaps because the larvae and adults must range more widely for food. Although *R. vespulae* can be controlled using pesticides, it is also possible to kill the larvae within infested materials through freezing or anoxia.

## 1. Introduction

*Reesa vespulae* (Milliron, 1939) belongs to the family Dermestidae. Since the mid-20th century, it has spread beyond its native habitats in North America [1], and is now often found in homes, warehouses and museums worldwide. The beetle represents an example of a continuing threat from biological invasions and the widening risk from beetles that attack stored products [2,3]. These skin beetles (or in German more distinctively Amerikanischer Wespenkäfer, the American wasp beetle) are considered as pests because they can damage stored products such as grain, cereal and dried food, but also animal hides. They can be a special problem in the heritage environment, as dermestids are often associated with animal materials such as leather and skin, wool, fur and dried insect collections, which makes natural history museums especially vulnerable [1,4,5]. The species was initially described from wasp nests in Minnesota as *Perimegatoma vesuplae* by Milliron in 1939. The beetle spread widely from the mid-20th century, with the first records of *R. vespulae* outside its native range arising from New Zealand in 1942 [6]. It was found in Europe through the 1950s [7,8,9]. In the UK, it was discovered in association with a grass seed warehouse in the 1970s [10], then in the Czech Republic [11] and most recently Bulgaria [12]. *Reesa vespulae* is currently prevalent throughout much of Europe, Australia, New Zealand, South America and North Africa [13]. It increased 20-fold in food warehouses in Germany in the 1980s, although the relatively slow rate of development meant that it was only a problem where seed was stored for more than a year [14]. However, restrictions on the range of allowed pesticides in the European Union has created some concern [11].

**Figure 1:**
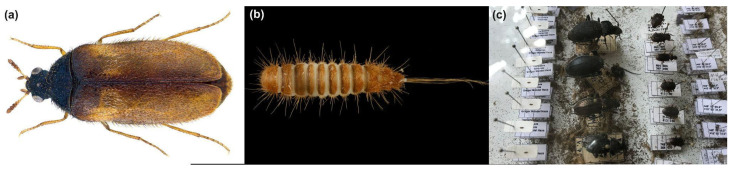
(**a**) Adult female *Reesa vespulae*—size: 2.9 mm, (**b**) larva and (**c**) damage to an entomological collection. Credits: (**a**) Adult beetle, kind permission of Udo Schmidt https://en.wikipedia.org/wiki/Reesa#/media/File:Reesa_vespulae_(Milliron,_1939)_(31097148261).png, accessed on 28 May 2024 (**b**) *R. vespulae* larva collected in Finland, Pekka Malinen, https://www.gbif.org/occurrence/4036519301 accessed on 28 May 2024 (licensed under http://creativecommons.org/licenses/by-sa/4.0/, 28 May 2024) (**c**) damage by *R. vespulae,* Pascal Querner.

A number of neobiotic animals have been of concern to heritage managers in recent years, including the rise of insects that damage heritage landscapes and the destructiveness of *Rhynchophorus ferrugineus* (Olivier, 1790), the red palm weevil [15]. There has additionally been an increase in termites that attack wooden structures or live plants or museum objects [15,16]. In European museums, a few species have been noted as spreading in recent years: *Attagenus smirnovi* (Zhantiev, 1973), the brown carpet beetle [17]; *Ctenolepisma calvum* (Ritter, 1910), the ghost silverfish [18]; *Gastrallus pubens* (Fairmaire, 1875) [19,20]; and *Thylodrias contractus* (Motschulsky, 1839), the odd or tissue paper beetle [21]. These seem to reflect a widespread and increasing problem for the heritage environment [22] (such as museums, libraries, art galleries and associated store rooms). *Reesa vespulae* is an important and potentially damaging pest for zoological and herbarium collections, with the larvae being especially destructive to these materials (Figure 1c). They are particularly common in entomological collections where they prefer Coleoptera or Lepidoptera [23]. The beetle was likely present in continental Europe from the 1960s, reaching Southern Finland (Tampere and Turku) and possibly Norway [24]. Today it has become part of a more general concern in museums internationally [23,25,26,27,28,29]. The beetle can be found among fluff and dust under furniture [30] or in the spaces beneath display cases. Such hidden locations may provide one of the reservoirs from which re-infestations develop [23]. *Reesa vespulae* can also grow on dead birds in roof areas [23], or can breed outside the museums in wasp nests [26].

As *R. vespulae* is a parthenogenetic species [1], and to date no males have been found, a single female can give rise to a new population, so pest control techniques based on mating disruption are not effective. This has raised concern about its ability to spread in museums from a remnant individual female [23,29]. The life cycle has been outlined by Bahr and colleagues [7,31] who observed that larvae develop at 25 °C over 1–1.5 years, at 23 °C over 2 years, and at 15–21 °C over 3 years. The adults live 6–14 days at room temperature, and after 2–3 days, they start laying 24 eggs on average. The eggs hatch as larvae typically after 3 weeks at 18 °C. There is no development below 13 °C. The long life cycle suggests that small increases in temperature could shorten this cycle and increase the abundance and the potential threat to collections under a warming climate.

In recent decades, there have been changes in pest management, and in European museums, it is likely that this has altered the presence and distribution of insect pests [32]. Concern about harmful pesticides has led to a more cautious approach to insect control under contemporary Integrated Pest Management (IPM) regimes [32]. This comes at a time when warmer conditions under a changing climate might shorten the insects’ life cycle or increase their activity [17,33]. There is an increasing international exchange of exhibitions among major museums, which can allow insects to travel with loaned natural history materials, especially objects associated with Hymenoptera, and additionally special exhibitions where the use of associated display materials can introduce new pests. In 2020, the COVID-19 pandemic led to the temporary closure of many museums and historic houses. This widened the range of habitats available to insects in museums at a time when there were few staff available to undertake IPM [34].

In early observations of the introduction of *R. vespulae* to Europe, it was evident that the insect posed a risk to museum collections [24]. This paper examines recent records of its increasing presence in the heritage environment, especially in Austria, but links it to observations from other European countries and elsewhere. Its growing presence in at least one Central European country, along with ready exchange and loan of exhibitions, means that there is a risk of further spread and infestation, especially in continental Europe. We explore recent catches from traps and observations that provide information about the increased potential for infestation.

## 2. Materials and Methods

This study benefited from insect monitoring that forms part of IPM procedures now adopted in many museums and historic libraries. The trapping data came from over 90 museums in Austria, which had continuous monitoring programmes that often began as early as 2014. The study examines the trapping records where *R. vespulae* were found, specifically from 17 buildings in Vienna and seven further afield from Lower Austria, Salzburg and Vorarlberg. At most sites, both sticky blunder traps (type Catchmaster) and pheromone traps (type Finicon) for webbing clothes moths were deployed. Traps were distributed at floor level at regular intervals along the edges of rooms and checked three to four times a year. In addition, some adventitious trapping took place in 2020–2021 (starting on 15 May 2020, with the last collection on 20 April 2021) at a small entomological collection at the University of Vienna. Small light traps were also used at this location in winter (L-trap, https://deffner-johann.de/de/l-trap-insektenfalle-klebefalle-mit-fotoluminiszens.html, accessed on 28 May 2024). Identification is relatively easy because *R. vespulae* is quite distinctive as an adult (Figure 1a), and even the larval form is quite characteristic (Figure 1b) and well described. The data are presented in Supplementary Table S1.

The study also used a number of datasets that reflect observations of catches of *R. vespulae*, in particular the Global Biodiversity Information Facility [35] and WhatsEatingYourCollection (WEYC) [16]. Data from the latter source are presented as deriving from a region rather than a specific museum, with limited details of the methods, but blunder traps are typically used. Data from three locations with catches of more than 20 records are presented in Supplementary Table S2. Personal contact with museum entomologists is also valuable in assessing the breadth of the threat from *R. vespulae*, and is mentioned in the text as personal communications or listed in the acknowledgements.

Statistical methods often adopt non-parametric approaches to reflect the integer nature of insect catches, so the Mann-Whitney test was used to compare catches from different locations. Additionally, results are reported as medians and variation as lower and upper quartiles (*Q*_1_ and *Q*_3_).

## 3. Results and Discussion

### 3.1. Overall Catch

**Figure 2:**
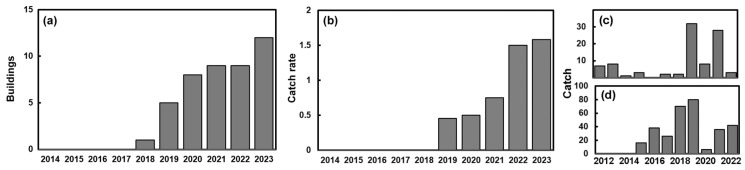
(**a**) The annual number of active buildings where *R. vespulae* was trapped, from the 24 buildings where the insect was found, although when the series began in 2014 there were only 21 buildings (see Table S1). (**b**) Average building catch for the sum of both adult and larval *R. vespulae* in the buildings in Austria. (**c**) Annual numbers of *R. vespulae* from WhatsEatingYourCollection (WEYC) data as reported from a museum in Birmingham and (**d**) annual numbers of *R. vespulae* reported from a museum store in Northern Ireland.

Figure 2a shows the change in the number of buildings where *R. vespulae* were caught in given years since 2014 from the Austrian monitoring programme. There is an increase in both the number of buildings where the beetle was caught along with an increase in the annual catch per building (Figure 2b). However, these results are rather biased, as a large proportion (⅓–⅔) of the total catch in some years comes from a single building and there are many instances where no beetles are caught. These observations arise from the 24 Austrian buildings out of 94 that are regularly monitored. There were 17 buildings in Vienna where 100 *R. vespulae* (both as adults and larvae) were caught. Additionally, traps were set for one year in a small entomological collection in the city. Outside Vienna, there were five buildings with *R. vespulae* and 17 examples where adults or larvae were caught. A Mann-Whitney test suggested that there was no difference (*p*_2_ > 0.5) in the catch numbers from the Viennese and non-Viennese buildings. In recent years (2022–2023), the catch from these Austrian heritage buildings was on average quite low, typically just over one insect per building (Figure 2b). However, including only those buildings where *R. vespulae* was found exaggerates the building catch (i.e., number caught per building), so even those buildings where *R. vespulae* was not caught need to be accounted for. Of the trapping programmes (many since 2014) involving the 94 different Austrian heritage buildings studied, only 25 have revealed any catch of *R. vespulae*, i.e., 30%. However, just recently (2024) an example was found in the library of Klosterneuburg Abbey, just north of Vienna. In the Austrian heritage buildings, on a year-by-year basis, there are 46 annual periods when the beetle was caught from a record that spans 630 building-years i.e., ~7%.

The proportion of museums where *R. vespulae* has been reported is a little lower elsewhere. In the UK, the WEYC database contains 367 different buildings, yet only 19 reports are associated with finds of *R. vespulae* (i.e., ~5%), and most of these are just a few isolated catches. If these data are examined on a year-by-year basis, which better accounts for the varying periods covered by the building records, the WEYC data, in terms of building-years, catches are found in 41 out of 1354 (3%). The WEYC data suggest that *R. vespulae* is reasonably well-known as a heritage pest in the UK although it is not necessarily reported often. There are strangely few records from London, where catches of other insects are frequently well recorded [36]. In Berlin, only one out of 20 buildings regularly investigated has a standing population of the beetle [personal communication, Bill Landsberger]. These percentages give a general impression that most museums are free of the pest, although we should emphasise that the frequency at which it is encountered in Austria appears to be higher than elsewhere. It seems that although it is not causing damage in Austria, *R. vespulae* has become more common over the last five years.

Despite the substantial fraction of Austrian museums reporting *R. vespulae*, none of these museums have especially large numbers, with only the Albertina, a collection of modern art, having annual catches into double digits. Here, *R. vespulae* was trapped from 2020–2023, so has persisted over a number of years. The most serious infestation occurred in a small entomological collection in Vienna, which suffered very badly from *R. vespulae*. It was not part of the regular monitoring programme and traps were only put out from spring of 2020 and set over the periods 15 March–22 July, 22 July–10 September and 10 September–20 April in the following year. The blunder traps only caught *R. vespulae* in the March–July period (41 examples). It is typically found in food warehouses in spring [37]. A light trap deployed at floor level over the entire period trapped 17 *R. vespulae*.

At three provincial locations in the UK, namely (i) Cardiff, (ii) the West Midlands (Birmingham) and (iii) County Down in Northern Ireland, there are more than 20 records of *R. vespulae*. Changes in catch over time from the West Midlands and County Down in Northern Ireland are shown in Figure 2c,d and suggest that the catch has been variable, but rather persistent. The average building catch each year for the museum in Birmingham over the years 2012–2022 was 8.5, but the catch numbers were highly skewed, so the central tendency might better be represented as the median, i.e., 3 (*Q*_1_ = 2; *Q*_3_ = 8). In Northern Ireland, the catch numbers were higher, with an average of 39.25 and median of 37 (*Q*_1_ = 23.5; *Q*_3_ = 49) for the years 2015–2022. They were even higher for the two years 2017–2018 in Cardiff, which averaged 89 (not plotted as a figure as the record was so short).

In Berlin, there has been a stable population since 2017, at only one location (out of almost 20), near a container harbour at Westhafen (personal communication, Bill Landsberger). In Bavaria, it is not found in any museum that has been monitored (personal communication, Stephan Biebl). Information from the south of France also suggests that *R. vespulae* is not very frequent (personal communication, Fabien Fohrer). In Norway, no special concern is apparent over changes in the populations of *R. vespulae* in heritage environments (personal communication, Anders Aak), while in Sweden, the insect has been found in natural history museums in both Stockholm and Malmö (personal communication, Niklas Apelqvist). Further afield in New Zealand, 43 adults, 52 larvae and 18 casings were discovered in nine locations in the Auckland Museum, between September 2017 and August 2018 [27].

### 3.2. Larvae and Adults

**Figure 3:**
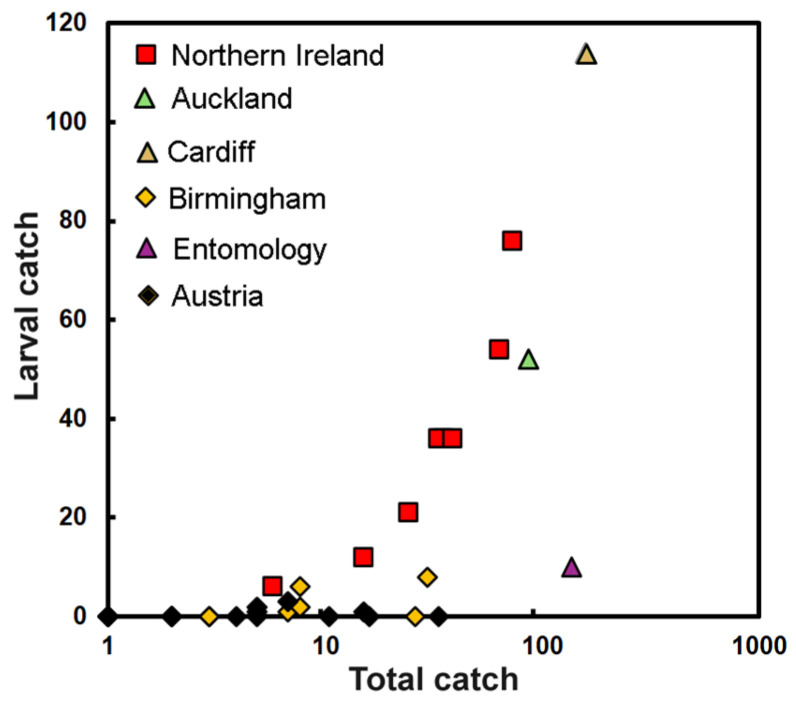
Annual number of *R. vespulae* larvae as a function of total catch at various buildings, with the exception of the Austrian samples, which are small and cover the length of the records, i.e., 7–9 years. Note: “Entomology” is from the small entomological collection in Vienna.

As seen in Figure 2b, the catch from Austrian heritage buildings was on average quite low: typically just one insect per building. Additionally, these were mostly caught as adults; only seven larvae were found in a total catch of 117 individuals. This represents just 6% of the catch. The low catch of larvae is illustrated in Figure 3, with all of the Austrian buildings lying close to the x-axis. A similar proportion was found for the year-long trapping at the Viennese entomological collection, where ten larvae were collected from a total of 152 individuals; thus, while large numbers of beetles were caught, larvae again represented only some 6% of the catch.

There was a proportionally greater presence of the larvae in the museum in Birmingham across the years 2012–2022, as larvae for the period averaged 18% of the total; however, as can be seen in Figure 3, the numbers varied year by year. The situation is very different in Northern Ireland, revealing especially high proportions of larvae from year to year and averaged to 88% of the total catch. The two years of data from Wales (2017–2018) showed that 64% were present as larvae, and data from the single year (2017–2018) of monitoring in Auckland showed a proportion of 55% [27]. These last three locations where the catch was high show much greater proportions trapped as larvae, as seen by points that appear to align themselves along a steeper trend. This was also true of an infestation in Natural History of the National Museum of Ireland from 2004, where a predominance of larvae (woolly bears) were trapped, although typically found under, rather than on, sticky traps [23].

Large infestations thus reveal more larvae, so it may be that they move away from the centre of infestations to avoid predation, but also gain more access to food resources, as both the adults and larvae are seen to range more widely.

### 3.3. Implications for the Heritage Environment

Trapping and monitoring museum pests is a key part of IPM. However, some have observed that a preoccupation with issues such as these may lead to failures in addressing changes in practice required to manage insect pest problems [38]. This makes it important to interpret the catch of pests in ways that are useful for treating the pests. Initially, it is important to interpret the data in terms of relevant metrics or pest occurrence indices [39,40]. It is of particular relevance to consider that Vaucheret and Leonard [23] reported that “individuals which were caught on the traps did not always match adequately with the areas where active infestations were discovered through visual inspections”. Thus, insect catches are not necessarily a reliable indicator, and regular visual inspections remain an important back-up.

There has long been a sense that problems with *R. vespulae* are increasing [25]. We have shown that although once unknown in Austrian museums, there are now around 20 that regularly report the beetle in low numbers. The global spread of *R. vespulae* is probably “the result of multiple introductions into the different zoogeographic regions, and secondary translocations therein” [1]. This means that a greater awareness of the insect is required. Changes in climate, a more globally derived visitor base and exchanges of collections from wider geographic areas could also contribute to increases in its presence. Changes in the layout of collections would be instances where it would be important to avoid transferring the insect [23]. Given the prevalence of damage to entomological collections with pinned insects [41], regular inspections of these seem especially important. More generally, it is also sensible to look within taxidermy and herbarium collections, along with vulnerable textiles. In Auckland Museum, *R, vespulae* were attracted to areas where staff store and consume food [27]. However, at the same time, it was noted that the beetle is attracted to dark, quiet collection spaces that are rarely accessed [27]. This could mean that museum storage areas are also vulnerable because of a lack of human activity. A growing proportion of larvae in larger catches might be a useful indicator for infestations of *R. vespulae*.

The levels of infestation, changes and their drivers over time are important to establish if a programme of eradication is planned. Once complete, continued awareness and regular inspection and cleaning is especially relevant in areas of current or earlier infestation [23].

Management of *R. vespulae* can take advantage of the long growth cycle and the high level of activity in spring. There is little evidence that the beetles fly in Austrian museums, as they do not appear to be associated with windows. Parry [42] found one specimen at a window in a Glasgow house, but did not observe it flying [personal communication, Ewan Parry]. Flight has been suggested under Czech conditions, as the beetle has been found in light-traps near a breeding site [43]. *Reesa vespulae* is a parthenogenetic species [1], and no males are found; therefore, a single female can give rise to a new population, and pest control techniques based on mating disruption are not effective. This raises concern about its ability to spread in museums from a remnant individual female [23,29].

*Reesa vespulae* can be controlled by freezing or anoxic environments. Arevad [44] suggested that the larvae of *R. vespulae* were killed on exposure to −20 °C. Bergh and colleagues [45] exposed beetle larvae in blocks of seasoned oak (20 cm × 20 cm × 20 cm) to −20 °C for 72 h, which ensured total mortality. Most were killed at temperatures some 5 °C warmer, but in materials such as wool these more modest degrees of freezing were less effective, so −20 °C may be the safer choice. In anoxic environments of almost pure nitrogen (i.e., oxygen below 1%), 99% of the *R. vespulae* larvae were killed after some 50 h [46]; similar results were shown more recently in other studies [47,48]. Pesticides such as pyrethrins, pyrethroids and organophosphates have been used in seed warehouses [11]. However, there is increasing reluctance to use pesticides in the heritage environment. Nevertheless, preventive strips with pyrethroids were used in the small entomological collection, but freezing has been more widely adopted in Viennese museums in places where objects have become infested.

## 4. Conclusions

The presence of *R. vespulae* in museums and libraries is widespread, although still comparatively rare in terms of the number of properties where it is found. Although it was recognised in 1970 that this insect posed a risk to museum collections, its presence in Austrian heritage environments has only become apparent over the last five years. However, with the exception of a small entomological collection, there has been little evidence of damage to collections in Austria. Nevertheless, its growing presence in one country in Central Europe, often in Natural History Museums and their storerooms, along with ready exchange and loan of exhibitions, means there is a risk of further spread and infestation. In other countries, the frequency of occurrence may be lower, but where this species occurs, the numbers trapped can be high and can lead to serious and damaging infestations.

The increasing observation of *R. vespulae* in museums suggests a need for greater vigilance. Trapping may not always catch the beetles, so visual inspections of likely habitats are important. Larvae are likely to be a good indicator of infestation and potential damage; although they have grown more common in Austria, infestations are rare and the larvae remain uncommon. As *R. vespulae* is a parthenogenetic species, the beetles can persist over years despite attempts to eradicate them. This has certainly raised concern that a remnant individual female can retain the ability to spread the insect within a museum. Additionally, work on the effectiveness of pesticide exposure may be useful, especially those that remain acceptable under modern IPM approaches.

The low frequency of occurrence has limited research in the museum environment, so more observations are needed. Research might consider that little is known about flight dispersal, seasonal behaviour and the resilience of populations under museum conditions. Given the rather lengthy life cycle, it may well be that in future warmer conditions, the cycle could be reduced from two years to one, potentially exacerbating the risk to vulnerable collections.

## Data Availability

The data from the traps are given in the Supplementary Materials, although the names of the heritage buildings have been anonymised.

## Supplementary Materials

The following supporting information can be downloaded at: https://www.mdpi.com/article/10.3390/insects15060405/s1, Table S1: Austrian data used; Table S2: Data from WEYC.
