# Supplementary material for: Catch of Reesa vespulae in Heritage Environments
Source: Insects. 2024 Jun 1;15(6):405. doi: 10.3390/insects15060405 (PMC11204326; doi:10.3390/insects15060405)
Supplement: Supplementary file 1 [file insects-15-00405-s001.zip › insects-3026640-supplementary.pdf]

Supplementary Tables

Table S1. Austrian data used

NM = no monitoring , - means no *Reesa vespulae*. l=larvae

	2014	2015	2016	2017	2018	2019	2020	2021	2022	2023
Vienna	-	-	-	-	4	4	2	1	4	2
Vienna	NM	NM	-	-	-	3	1	1	0	9+1 l.
Vienna	-	-	-	-	-	1	-	-	-	-
Vienna	-	-	-	-	-	1	1	1	1	-
Lower Austria	-	-	-	-	-	1	-	-	-	-
Entom.*	NM	NM	NM	NM	NM	NM	41	NM	NM	NM
Vorarlberg	-	-	-	-	-	-	2	3	5	1
Vienna	-	-	-	-	-	-	1	-	-	-
Vienna	-	-	-	-	-	-	2	-	-	-
Vienna	-	-	-	-	-	-	1	-	-	-
Lower Austria	-	-	-	-	-	-	1	-	-	2+1 l.
Vienna	NM	NM	NM	NM	NM	NM	NM	6	21	9
Salzburg	-	-	-	-	-	-	-	1	1	-
Lower Austria	-	-	-	-	-	-	-	1 + 2 l.	-	-
Vienna	-	-	-	-	-	-	-	1	-	-
Vienna	-	-	-	-	-	-	-	1	-	-
Vienna	-	-	-	-	-	-	-	-	1+1 l.	2 l.
Vienna	-	-	-	-	-	-	-	-	1	-
Vienna	-	-	-	-	-	-	-	-	1	?
Vienna	-	-	-	-	-	-	-	-	-	1

Vienna	-	-	-	-	-	-	-	-	-	1
Vorarlberg	-	-	-	-	-	-	-	-	-	5
Vienna	-	-	-	-	-	-	-	-	-	1
Vienna	-	-	-	-	-	-	-	-	-	1
Vienna	NM	NM	NM	NM	NM	NM	NM	-	-	2

*The small entomological collection in Vienna is for a single year so not included in the general analysis.

Table S2. Data from WEYC

	West Midlands	County Down	Cardiff
2012	7		
2013	8		
2014	1		
2015	3	16	
2016	0	38	
2017	2	26	136
2018	2	70	42
2019	32	80	
2020	8	6	
2021	28	36	
2022	3	42	
